# Role of microRNA-183 based theranostics through targeting TPM1 in bladder cancer

**DOI:** 10.1007/s12672-025-03829-w

**Published:** 2025-11-13

**Authors:** Samah Mamdouh, Tarek Aboushousha, Eman Hemida, Rady E. El-Araby, Khaled Elesaily, Gehan Hammad, Mona Magdy

**Affiliations:** 1 Biochemistry and Molecular Biology Department, Theodor Bilharz Research Institute, Giza, Egypt; 2 Pathology Department, Theodor Bilharz Research Institute, Giza, Egypt; 3 Biochemistry Department, Obstetrics and Gynecology Hospital, Faculty of Science, Ain Shams University, Cairo, Egypt; 4 Department of Basic and Clinical Translation Sciences, Tufts University School of Dental Medicine, Boston, MA 02111 USA; 5 Central Lab, Theodor Bilharz Research Institute, Giza, Egypt; 6 Urology Department, Theodor Bilharz Research Institute, Giza, Egypt; 7 Faculty of Biotechnology, October University for Modern Sciences and Arts, Giza, Egypt

**Keywords:** MicroRNA, Bladder carcinomas, TPM1, miR-183-5p, Theranostic, Biopsy, Urine cytology

## Abstract

**Background:**

MicroRNA-183 (miR-183-5p), a noncoding RNA, is upregulated in bladder carcinoma (BC). Although it has been implicated in oncogenesis, its precise regulatory effects and biological functions remain unclear. Tropomyosin-1 (TPM1) was shown to be downregulated in solid tumors and was previously identified as a novel tumor suppressor gene.

**Objectives:**

Our study focuses on the prognostic, diagnostic, and therapeutic potential of miR-183-5p in bladder carcinoma and assess TPM1 gene targets and their modulatory functions.

**Methods:**

Urine cytology, cystectomy and transurethral resection (TUR) biopsies from 148 BC patients were collected. TPM1 protein and miR-183-5p expressions were assessed through immunohistochemistry (IHC) and real-time PCR respectively. In vitro assay investigated the effect of miR-183-5p on TPM1mRNA in bladder carcinoma cell lines, then confirmed by comparing miR-183-5p mimics in non-cancerous and urothelial carcinoma cell lines. Concomitant TPM1 gene expression was examined, and the theranostic miR-183-5p potential was evaluated.

**Results:**

Upregulation of miR-183-5p expression in BC tissue biopsies and urine cytologies was noted, contrasting the downregulation of TPM1 protein expression in the high-grade, high-stage, lymph node metastatic BC tissues (in comparison to non-cancerous, low-grade, low-stage non-lymph node metastasizing BCs). Moreover, the miR-183-5p oncogenic influence was responsive in targeting TPM1 gene at 3′UTR region, and miR‑183‑5p.1 restricted TPM1 expression in T24 cells.

**Conclusion:**

This study provides the first illustration of the miR-183-5p–TPM1 axis in bladder carcinoma, supporting the theranostic role of miR-183-5p as an onco-miR in BC progression, diagnosis, and prognostication.

## Key points

- Upregulation of miR-183-5p: The study demonstrates significant upregulation of miR-183-5p in bladder carcinoma tissues and urine, especially in high-grade and metastatic cases, suggesting its potential as a non-invasive biomarker for BC diagnosis.Upregulation of miR-183-5p: The study demonstrates significant upregulation of miR-183-5p in bladder carcinoma tissues and urine, especially in high-grade and metastatic cases, suggesting its potential as a non-invasive biomarker for BC diagnosis.
- Urine-based Detection: miR-183-5p was effectively detected in urine samples, showing potential for early detection of BC through a non-invasive method, improving upon current diagnostic tools like cytology and cystoscopy.
- Tumor Suppressor Role of TPM1: The study confirms that TPM1 is significantly downregulated in BC, particularly in high-grade, muscle-invasive, and metastatic bladder cancers.
- Inverse Relationship: There is an inverse correlation between miR-183-5p expression and TPM1 levels, with miR-183-5p directly targeting TPM1 and suppressing its expression, contributing to cancer progression.
- Theranostic Role of miR-183-5p: This study positions miR-183-5p not just as a diagnostic marker but also as a potential therapeutic target (theranostic) that can be manipulated to inhibit bladder cancer progression.
- miR-183-5p and Disease Severity: The expression of miR-183-5p correlates with tumor grade, stage, and metastasis, making it a reliable marker for prognostication. High miR-183-5p levels are associated with worse clinical outcomes.

## Introduction

In 2020, Egypt recorded the highest mortality from bladder carcinoma (BC) in the Middle East [1, 2] BC is classified as non-muscle-invasive (NMIBC) or muscle-invasive (MIBC) [3]. At the first diagnosis, almost 70% of patients present as NMIBC, 50%–70% relapse and 10%–20% progress to MIBC [4], which is more likely to metastasize and correlates with high mortality rates despite the currently advanced therapeutic regimens [5, 6]. In this regard, prediction of models that can identify an unfavorable prognosis for patients, particularly those who may benefit from the early diagnosis and therapy, is greatly required.Urinary cytology and cystoscopy are the front-line approaches to identify and diagnose BC [7]. Moreover, the development of innovative non-invasive biomarkers, that are readily available, stable in body fluids, and can reflect clinical–pathological features of the disease [8] would be tests of choice, particularly for urine screening, for dramatic improvement of tumor detection accuracy and reduction of progression and BC recurrence risks. In this regard, urine non-invasive screening tests, which are based on cytology, have become the focus of recent research [9]. Nevertheless, there is still a deficiency in sensitive and specific biomarkers that can distinguish different BCs subtypes. MiRNAs, are endogenous noncoding RNAs, and as such have become an emphasis in research pertaining to disease identification [10, 11]. They were previously demonstrated to regulate gene expression changes by mRNA targeting; hence, they presenting roles in cell growth, differentiation, functional changes within cells marking their involvement in disease progression/inhibition [12], For cancers they function in either the promotion or suppression of tumor growth and metastasis; thus playing an important role in tumor progression, particularly for BCs [13, 14]. MiRNAs can be readily isolated from bodily fluids such as the urine and serum of BC patients with minimal invasiveness [15]. In this regard, urinary miRNAs represent an attractive biomarker based screening tool, which has an evident role in genitourinary cancer urine detection and constitute a novel approach for BC assessment and prognostication [16]. Therefore, identifying potential molecular targets is crucial for BC identification and treatment. MicroRNA-183-5p (miR-183-5p) was previously reported to have an involvement in several cancer hallmarks such as tumorigenesis and metastasis, and is located on chromosome 7q32.2 [17, 18], , several cancer types were studied for a correlation to this miRNA including gastric cancer [19, 20] and pancreatic adenocarcinomas [21]. Although miR-183-5p’s oncogenic effect targets Tropomyosins (TPMs), through a TPM-related receptor kinase B (TrkB), it promotes invasion and metastasis, and indicates poor prognosis in various malignancies [22]; yet based on current literature findings this role has not been explored in urinary bladder cancers. Tropomyosins (TPMs), are actin-connecting proteins, that are expressed in non-muscle cells, and exhibit a critical role in stress fibers and actin cytoskeleton modulation. In tumor cells, they enhance migration and invasiveness through stress fibers destruction by TPM-mediated pathways [23, 24]. Tropomyosin was noticed as a promising BC diagnostic and prognostic marker [6]. Nevertheless, the mechanism of TPMs in BC remains ambigious. Therefore, using miR-183-5p to target the TPM isoform 1 (TPM1) gene may help as a potential therapeutic and management strategy in BC patients. To our current knowledge, there were no previous describing the relationship between miR-183-5p and TPM1 in BC. This study aims to determine the diagnostic and therapeutic potential of miR-183-5p in bladder carcinomas by targeting TPM1 gene, through estimating the miR-183-5p expression profiles in urine cytology and their corresponding BC biopsies, with subsequent TPM1 protein expression evaluation in urinary bladder biopsies.

## Methods

### Plan of work

The expression and the clinical significance of miR-183-5p was a primary objective in this study and thus it was assessed in both urine cytology samples and bladder tissue biopsies. Subsequently, the prediction of miR-183-5p target genes was conducted through TargetScan bioinformatics tool. Then an in vitro study for miR-183-5p to target the predicted TPM1 gene was performed. Finally, the TPM1 protein immunohistochemical (IHC) expression in bladder tissue biopsies was assessed for all the studied groups.

### Patients

This study included a total of 178 urology patients (including 148 with BC and 30 with cystitis only non-BC patients as control), who visited the outpatient clinic of Theodor Bilharz Research Institute hospital (TBRI), Giza, Egypt. Before recruitment, an informed consent was signed by the patients. According to the guidelines of the ethical principles, as outlined in the Declaration of Helsinki, the research protocol was approved by the institutional review board of the TBRI Ethics Committee, under the Federal Wide Assurance No. FWA00010609, and in accordance with the institutional guidelines. Urine samples and urinary bladder tissue biopsies (cystectomies or Transurethral resection of bladder tumor (TUR-BT) were obtained.

### Urine samples and biopsies preparation

Urine samples (50 ml) were collected from all participants and subjected to centrifugation at (3000 rpm) for 20 min. Pellets were stored at (− 80 °C) following the decanting of the supernatant pending RNA extraction. Simultaneously, and after smears staining with Papanicolaou’s (PAP), microscopic urine cytology examination was performed according to The Paris System for reporting urinary cytology [25]. A positive cytology was acknowledged when at least 5 High Grade Urothelial Carcinoma (HGUC) nuclei exhibited a high N/C ratio (>0.7, as nucleus occupied >70% of cytoplasm), together with hyperchromasia, irregular nuclear membranes pleomorphism, or abnormal chromatin pattern. Biopsies obtained from all cases for the immunostaining evaluation, were fixed in (10%) formalin, processed into paraffin sections, and pathologically evaluated for tumor type, stage, grade, and lymph node metastasis by 2 independent pathologists. The stage of the patients was determined according to the TNM staging system of the American Joint Committee on Cancer classification system.

### Cell culture

Culturing of T24 BC human cell line and the SV-HUC-1 normal urothelial cell lines sourced from Tissue culture unit at Theodore Bilharz Research Institute (TBRI), were conducted at (37˚C), with (5%) CO_2_, in T25 culture flasks. The T24 human cell line was cultured in Dulbecco’s Modified Eagle’s Medium (DMEM), with supplementation of (10%) Fetal Bovine Serum (FBS) (v/v), 100 U/ml Penicillin and 100 µg/ml Streptomycin, while the SV-HUC-1 cell line was cultured in F12K medium, with 10% FBS. The morphology of cells was confirmed using an inverted microscope periodically and passaged at confluence (80%) until the cell lines were established and stably dividing.

### Transfection of miR-183-5p.1

T24 and SV-HUC-1 cells were seeded in 6-well plates, at 50% confluence, were incubated at 37˚C overnight. After 24 h, cells were transfected with high (100 nM) and low (30 nM) doses of miR-183-5p.1 mimics, with Lipofectamine 2000™ (Invitrogen; Thermo Fisher Scientific, Inc.) as a delivery system, according to the manufacturer’s instructions. Untransfected cells, were considered as control group. The medium was then changed to a complete medium, after 24 h of subjecting the cells to transfection medium. Then, Tropomyosin expression was quantified using RT-qPCR.

### MicroRNA extraction from urine samples and tissue biopsies

miR-183-5p and TPM1 primers were manufactured by Invitrogen (Thermo Fisher Scientific, Inc.). For urine samples, miRNA-183-5p was isolated from urine pellets and tissue samples using mirVana Kit^®^ (Applied Biosystems, CA, USA). For each tissue biopsy, 100 mg of BC fresh tissues was used after homogenization in a stainless-steel mortar and chilling on dry ice. The concentration and purity were determined by a Nanodrop^®^ ND-1000 spectrophotometer (Thermo Fisher Scientific). While total RNA was isolated from cultured cells using TRIzol reagent (Thermo Fisher Scientific, Inc.) and was also quantified spectrophotometrically.

### Quantitative real-time reverse-transcription assay (qRT-PCR)

miR-183-5p TaqMan primer assays and probes and the TPM1 primers were acquired from Applied Biosystems, San Diego, CA. RT-qPCR assays were done according to the manufacturer’s instructions, details of these primers are shown in Table 1 [20]. The cDNA was synthesized out of 5 ng of total RNA using the Taqman miRNA reverse transcription kit (Applied Biosystems, USA). Briefly, 5 µl cDNA was used for the amplification step. The reaction was conducted at 95 °C, for 10 min, then 40 cycles of 95 °C, for 15 s, and 60 °C for 60 s, using the StepOne™ Real-Time PCR System (AB Applied Biosystems, Foster City, CA, USA). All reactions were done in duplicates. GAPDH and U6 genes were used as the internal controls. The miRNA expression was determined based on the threshold cycle (Ct), and the relative expression level was determined using 2^-[(Ct of miR-183−5p)–(Ct of U6)]^ after normalization with the U6 small nuclear RNA expression according to Yilmaz et al. [26], in all samples.

**Table 1:** Sequence of primers used for RT-qPCR

Gene	Forward Primer Sequence (5’-3’)	Reverse (5’-3’)
TPM1	GCCGACGTAGCTTCTCTGAAC	TTTGGGCTCGACTCTCAATGA
GAPDH	ATTCCATGGCACCGTCAAGGCTGA	TTCTCCATGGTGGTGAAGACGCCA
miR-183-5p	TATGGCACTGGTAGAATTCACT	GCGAGCACAGAATTAATACGAC
U6	CTCGCTTCGGCAGCACA	AACGCTTCACGAATT TGCGT

### Immunohistochemical assessment of TPM1 protein in bladder biopsies

Polyclonal anti-TPM1 protein antibody (Cat. Number: YPA2403, Chongqing Biopsies, China) was used for immunohistochemistry (IHC) labelling of tissue sections cut from paraffin blocks at 4 μm onto positively charged slides (Super Freeze Plus, Menzel-Glaser, Germany). Following that, the slides were stained using the Dako Auto-Stainer Link 48 automated platform at a dilution of 1:100. Heat-induced antigen retrieval was done for 30 min at 97 °C using the high-PH En Vision™ FLEX Target Retrieval Solution. The slides were blocked with inhibitor D (3% H_2_O_2_) for 4 min at 37 °C before being treated with the primary TPM1 antibody for 40 min at 37 °C and the universal secondary antibody for 20 min at 37 °C. Finally, the slides were treated with streptavidin-horseradish peroxidase (SA-HRP) D for 15 min, at 37 °C, followed by the DAB substrate (3,3′-diaminobenzidine tetrahydrochloride), then H_2_O_2_ for 10 min, and counter-stained with Hematoxylin.

### Semi-quantitation of IHC staining

Positive cells percentage (PP%) and the staining intensity (SI) were semi-quantitatively evaluated. SI was calculated as follows: 0 = negative staining, 1 = weak staining; 2 = moderate staining; and 3 = strong staining. PP% was calculated as follows: 0 = no positive cells; 1 if < 10% positive cells, 2 = 10–50%, 3 = 51–80%, and 4 if >80% positivity in cells. According to Venerito et al. [27], , the Immunoreactive Score (IRS) was calculated by 2 independent pathologists, as PP x SI, reaching a maximum of 12, and then an average score was considered.

### Statistical analysis

For data analysis Microsoft Excel 2016 as well as SPSS version 26 were employed. Mean ± SD represented the normal continuous variables at a 95% confidence interval. The 25% and 75% summarized the nonnormal variables, and *P* < 0.05 was considered statistically significant. Student’s t-test was used to compare the normal variables means between groups. Mann-Whitney U test was performed for non-normal variables. χ^2^ test or Fisher’s exact test determined the categorical variable distributions between the groups. The Receiver Operating Characteristic curves (ROC) assessed the diagnostic performance of the markers. The area under the curve (AUC) was calculated as a prognostic accuracy index together with the univariate analysis.

## Results

### Bioinformatic analysis of the target genes of miR-183-5p

TargetScan was utilized for the prediction of miRNAs’ biological targets by identifying the presence of 6-mer, 7-mer, and 8-mer sites that match each miRNA seed region and as an option [28], only conserved sites were predicted. Moreover, the mismatch sites in the seed region which were compensated by the conserved 3’ pairing and the centered sites [29]were identified. In mammals, predictions were ranked based on the predicted efficacy of targeting, using cumulative weighted contexts plus the score of the sites [30], and by their probability of conserved targeting PCR [31], as an option, and within the open reading frames (ORFs). The human TargetScan considers the matches to the human’s 3’ UTRs, according to the UCSC whole-genome alignments [32]. In the present study, TargetScan’s Software predicted the TPM1 gene as a potential target for miR-183-5p.1 and miR-183-5p.2, according to its mRNA 3′-UTR untranslated region, complementary to the miR-183-5p (Table 2).

**Table 2:** Prediction of the prospective target genes of miR-183-5p. By targetscan

	Predicted consequential pairing of the target region (top) and miRNA (bottom)	Site type	Context + + score	Context + + score percentile	Weighted context + + score	Conserved branch length	*P*_CT_	Predicted relative K_D_
Position 193–199 of TPM1 3’ UTR hsa-miR-183-5p.1	5’ …GAUCCUGGUUCAAAU-GUGCCAUU… ||| ||||||| 3’ UCACUUAAGAUGGUCACGGUAU	7mer-m8	-0.41	98	-0.41	3.806	0.46	-5.160
Position 299–306 of TPM1 3’ UTR hsa-miR-183-5p.2	5’ …AGAAGUUCCAUUCAA–AGUGCCAA… |||| ||||||| 3’ GUCACUUAAGAUGGUCACGGUA	8mer	-0.68	99	-0.68	3.479	0.35	-5.666

### The association between the miR-183-5p.1 and TPM1 in the in vitro BC model

**Fig. 1:**
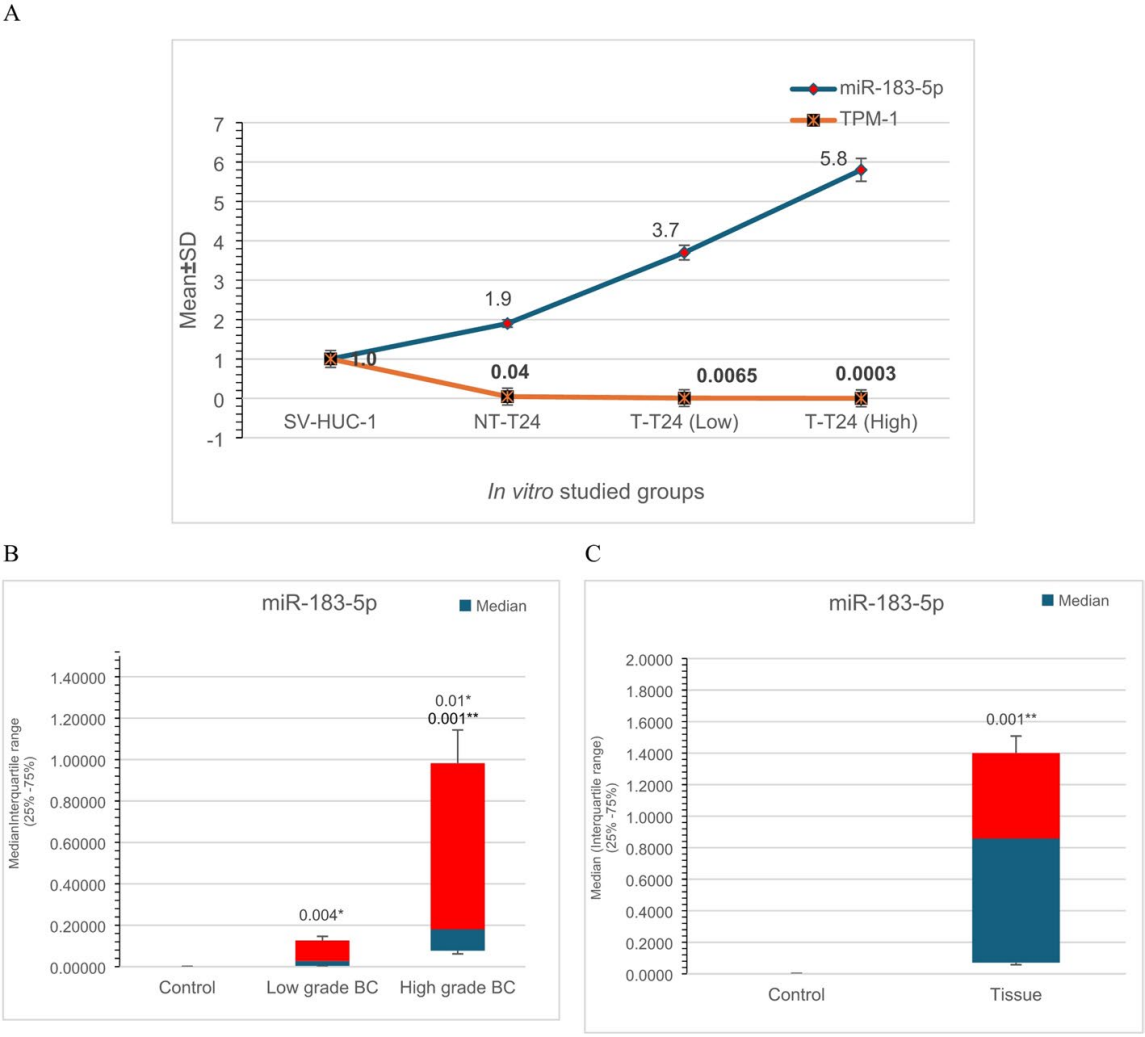
Relative expression of the miR-183-5p and TPM1 gene among the studied cases. A: Relative miR-183-5P and TPM1 gene expression in SV-HUC-1 and T24 cell lines. miR-183-5P and TPM1. Gene expression was calculated with triplicates for the fold-change, while the fold-change was calculated according to 2^−ΔΔCT^. Fold change values less than one indicate downregulation, whereas values greater than one indicate upregulation. (*n* = 3, student t-test, ^**^P < 0.01 vs. SV-HUC-1 and ^##^*P* < 0.01 vs. non-transfected cells (NT-T24). B: Relative expression of miR-183-5p in the urine samples; non-cancer control, low grade, and high-grade urine of BC. Relative miR183-5p expression was represented as Median (Blue part) and interquartile range (25% -75%) (Red part) of the fold-change of the studied groups. C: Relative expression of miR-183-5p in urinary bladder biopsies. Relative miR183-5p expression was represented as Median (Blue part) and interquartile range (25% -75%) (Red part) of the fold- change of the studied groups. P value < 0.001 is highly significant. Gene expression was calculated with triplicates for the fold change, while the fold-change was calculated according to 2^−ΔΔCT^. Fold change values less than one indicate downregulation, whereas values greater than one indicate upregulation

To prove this relationship, the transfection with miR-183-5p.1 mimics has led to a marked downregulation of TPM1 gene expression in the T24 cell lines at the mRNA level, while our results identified a 0.04-fold difference in non-transfected T24 (NT-T24) when compared with SV-HUC-1 normal cell lines (*P* < 0.001). Significantly, the transfected T24 with miR-183-5p.1 mimics exhibited 0.0065- and 0.0003-fold differences when transfected at low and high doses respectively (*P* < 0.001), in comparison to SV-HUC-1 and NT-T24 cell lines (Fig. 1A). Interestingly, the expression of miR-183-5p shows a significant correlation with TPM1 gene expression; thus, suggesting that miR-183-5p as an onco-miR is involved in the TPM1 gene expression and regulation in BCs cells. Taken together, our results indicated that the TPM1 gene is a target for miR-183-5p and represented a mechanism for tumorigenesis modulation by miR-183-5p.

### Clinicopathological parameters correlation

In our study, 178 urine cytology and their corresponding biopsies (whether cystectomy or TUR) were examined (including 148 BCs and 30 non-cancer cystitis cases as control). Overall, the study revealed a significant difference between high and low-grade bladder carcinoma regarding sex of the patients (*P* = 0.03). The presence of High-Grade Urothelial Carcinoma (HGUC) cells was associated with a positive urine cytology, bilharziasis, muscle invasion, the higher tumor stages (T2 & T3), presence of lymph nodes metastatic deposits and GII, GIII tumor grades (*P* = 0.001), compared to the lower-grade tumors (Table 3).

Additionally, a significant association was observed between the miR-183-5p expression in BC tissues and patients with high serum creatinine (*P* = 0.001).

**Table 3:** Clinicopathological features and characteristics of bladder carcinoma patients

Parameter		Total patients *N* = 148	Bladder carcinoma patients		
			Low grade BC *N* = 52	High grade BC *N* = 96	*P*. value
Age		59.5 ± 7.6	58.9 ± 6.9	60.2 ± 8.3	0.8
Sex	Female	16(10.8%)	8(15.4%)	8(8.3%)	0.03*
	Male	132(89.2%)	44(84.6%)	88(91.7%)	
Smoking	No	28(18.9%)	12(23.1%)	16(16.7%)	0.09
	Yes	120(81.1%)	40(76.9%)	80(83.3%)	
bilharziasis	Negative	48(32.4%)	28(53.8%)	20(20.8%)	0.001**
	Positive	100(67.6%)	24(46.2%)	76(79.2%)	
Serum creatinine	Normal	124(83.8%)	52(100.0%)	72(75.0%)	0.001**
	High	24(16.2%)	0(0.0%)	24(25.0%)	
Clinical staging	Ta	24(16.2%)	20(38.5%)	4(4.2%)	0.001**
	T1	36(24.3%)	28(53.8%)	8(8.3%)	0.001**
	T2	48(32.4%)	4(7.7%)	44(45.8%)	0.001**
	T3	40(27.0%)	0(0.0%)	40(41.7%)	0.001**
Urine cytology	Negative HGUC	72(48.6%)	48(92.3%)	24(25.0%)	0.001**
	Positive HGUC	76(51.4%)	4(7.7%)	72(75.0%)	
Lymph node metastasis	Negative	96(64.9%)	52(100.0%)	44(45.8%)	0.001**
	Positive	52(35.1%)	0(0.0%)	52(54.2%)	
Histopathological characteristics	Squamous cell carcinoma	20(13.5%)	0(0.0%)	20(20.8%)	0.08
	Transitional cell carcinoma	128(86.5%)	52(100.0%)	76(79.2%)	
Pathological Tumor Stage	Negative	36(24.3%)	28(53.8%)	8(8.3%)	0.001**
	Ta	28(18.9%)	24(46.2%)	4(4.2%)	0.001**
	Non-muscle invasive	20(13.5%)	0(0.0%)	20(20.8%)	0.01*
	Muscle invasive	64(43.2%)	0(0.0%)	64(66.7%)	0.001**
Tumor Grade	GI	52(35.1%)	48(92.3%)	4(4.2%)	0.001**
	GII	28(18.9%)	0(0.0%)	28(29.2%)	0.001**
	GIII	68(45.9%)	4(7.7%)	64(66.7%)	0.001**

BC: Bladder carcinoma. HGUC: High Grade Urothelial Carcinoma. Age is presented as Mean ± SD, which was analyzed by student’s t-test. While Sex, Bilharziasis, Smoking status, Serum creatinine, Clinical Ex., Lymph node, Cytology, Histopathological characteristics, Tumor stages, and tumor Grades are presented as F (%) frequency and percent; the data were analyzed by X2 test. * P value < 0.05 is significant, **P value < 0.001 is highly significant

### Expression profiling of miR-183-5p in urine samples

Using U6 as a miRNA reference and Mann-Whitney U test to statistically analyze the data, our results elucidated a significant upregulation of miR-183-5p expression levels in the urine of BC patients when compared to non-cancerous cystitis (controls) with median and interquartile range 25% -75% = 0.0003(0.00006–0.0005), 0.03(0.0008–0.16) and 0.03(0.0008–0.16) for control urine samples, low grades and high grades respectively. Moreover, its expression proportionated directly and significantly with the tumor grades. In this regard, the analysis illustrated that miR-183-5p can significantly distinguish the controls from the low grade tumors in urine (*P* = 0.004), and the controls from the high grade tumors (*P* = 0.001). Furthermore, it can significantly differentiate low grade from high grade BC in urine (*P* = 0.01) (Table 4; Fig. 1B).

**Table 4:** miRNA-183-5p level in urine samples

Urine	Control *N* = 30	Low grade BC *N* = 52	High grade BC *N* = 96	*P*. value		
				Low grade vs. Control	High grade vs. Control	High grade vs. Low grade
miR-183-5p	0.0003(0.00006–0.0005)	0.03(0.0008–0.16)	0.03(0.0008–0.16)	0.004**	0.001**	0.01*

The fold change results are according to (2^−∆∆CT^). All parameters are represented as Median with Interquartile range (25% -75%) of the fold change of the studied groups, the data were analyzed by the Mann-Whitney U test. ^*^ P value < 0.05 is significant, ^**^P value < 0.001 is highly significant. BC: Bladder cancer

### Expression profiling of miR-183-5p in bladder biopsies

Synchronously, the analysis showed significantly high miR-183-5p expression levels in BC biopsies when compared to non-cancerous cystitis tissues with median and interquartile range 25% -75% = 0.002(0.0004–0.003) and 0.86(0.07–1.57) for controls and BC tissues respectively with *P* = 0.001, (Table 5; Fig. 1C). Nevertheless, there was no statistically significant association between miR-183-5p levels in BC tissues and either tumor stage, lymph node metastasis, or positivity for HGUC in urine cytology.

**Table 5:** miRNA-183-5p level in urinary bladder tissue biopsies

Parameter	Control *N* = 30	BC Tissue *N* = 148	*P*. value
miR-183-5p	0.002(0.0004–0.003)	0.86(0.07–1.57)	0.001**

The fold change results are according to (2^−∆∆CT^). All parameters are represented as Median with Interquartile range (25% -75%) of the fold change of the studied groups, the data were analyzed by the Mann-Whitney U test. ^*^ P value < 0.05 is significant, ^**^P value < 0.001 is highly significant. BC: Bladder cancer.

### Diagnostic performance of miR-183-5p in urine samples

**Fig. 2:**
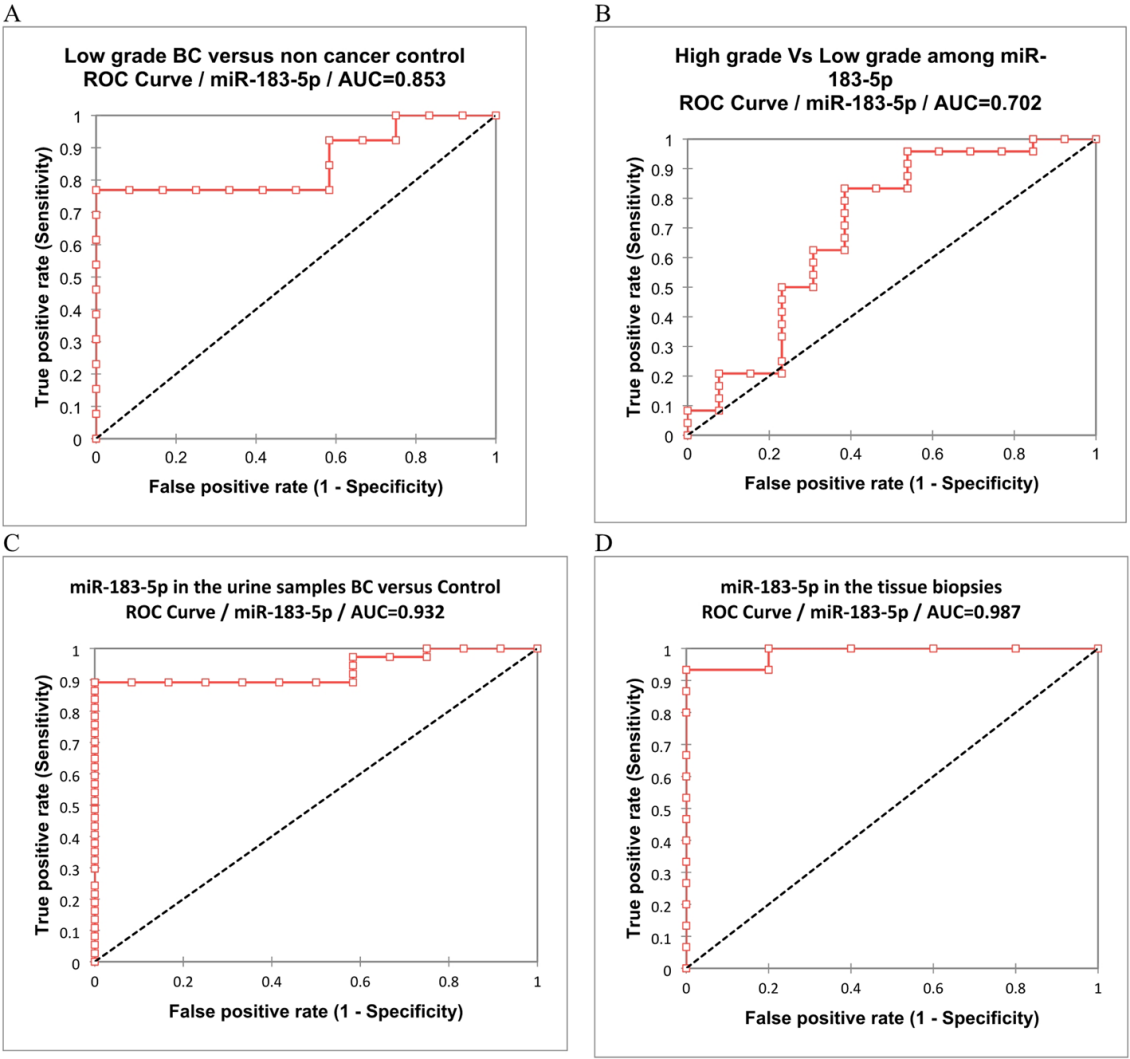
Assessment of miR-183-5p diagnostic performance in discrimination Bladder Cancer (BC) progression among the studied cases. **A**: Low grade BC vs. non-cancer control in urine cytology. **B**: High grade vs. low grade BC in urine cytology. **C**: Overall miR-183 discriminatory performance in the urine cytology. **D**: Overall miR-183-5p discriminatory performance in bladder tissue biopsies

In our study, we used ROC Curve to analyze the miR-183-5p diagnostic performance in urine samples of BC patients, to assess the sensitivity and specificity for BC prediction, and to illustrate their discriminatory power between cancerous and non-cancerous urine. Our data revealed a significant discrimination between low grade BC versus (vs.) non-cancer controls, at miR-183-5p cut-off value of 0.002, with 76.9% sensitivity, 100% specificity, 100% positive predictive value (PPV), 80% negative predictive value (NPV), 88% accuracy, 0.853 Area Under Curve (AUC) and 95% C.I = (0.691-1) with (*P* < 0.0001) (Table 6; Fig. 2A). Similarly, the statistical analysis elucidated a significant discrimination between high grade vs. low grade BCs at miR-183-5p at cut-off value of 0.053, with 83.3% sensitivity, 61.5% specificity, 80% PPV, 66.7% NPV, 75.7% accuracy, 0.702 AUC, 95% C.I= (0.503-0.9) with (*P* = 0.04) (Table 6; Fig. 2B).

**Table 6:** Cut-off values for the diagnostic performance of miR-183-5p in urine samples

Urine	Cut-off	Sensitivity	Specificity	PPV	NPV	Accuracy	AUC	95% C. I		*P*. value
								Lower	Upper	
Low grade BC Vs non-cancer Control	0.002	76.9%	100.0%	100.0%	80.0%	88.0%	0.853	0.691	1.000	< 0.0001**
High grade Vs Low grade BC	0.053	83.3%	61.5%	80.0%	66.7%	75.7%	0.702	0.503	0.900	0.04*

BC: Bladder carcinoma, PPV: Positive predictive value, NPV: negative predictive value, AUC Area under the curve, and C.I: 95% Confidence Interval. * P value < 0.05 is significant, ** P value < 0.001 is highly significant.

### Diagnostic performances of miR-183-5p in tissue biopsy samples compared to urine cytology

In urine cytology, our data exhibited that miR-183-5p significantly diagnosed BC at a cut-off = 0.002, with 89.2% sensitivity, 100% specificity, 100% PPV, 75% NPV, 91.8% accuracy, 0.932 AUC, 95% C.I =(0.864-1) with *P* < 0.0001 (Table 7; Fig. 2C). Likewise in bladder biopsies, miR-183-5p significantly diagnosed BC at the cut-off = 0.004, with 93.3% sensitivity, 100% specificity, 100% PPV, 83.3% NPV, 95% accuracy, and, 0.987 AUC, 95% C.I = (0.961-1) with *P* < 0.0001 (Table 7; Fig. 2D).

**Table 7:** Overall diagnostic performance of miR-183-5p in urine cytology and tissue biopsies

Sample Type	Cut-off	Sn. %	Sp. %	PPV %	NPV %	Accuracy %	AUC	95% C. I		*P*. value
								Lower	Upper	
Urine	0.002	89.2	100	100	75	91.8	0.932	0.864	1.000	< 0.0001**
**Tissue**	**0.004**	**93.3**	**100**	**100**	**83.3**	**95.0**	**0.987**	**0.961**	**1.000**	< 0.0001**

PPV: Positive predictive value, NPV: negative predictive value, AUC Area under the curve, and C.I: 95% Confidence Interval. * P value < 0.05 is significant, ** P value < 0.001 is highly significant.

### Univariate and multivariate logistic regression analysis

Regarding BC prediction, our analysis revealed statistical significance in BC urine samples contrasting bladder tissue biopsies. To clarify, a 1-degree increase in miR-183-5p in urine resulted in significant increase by a factor of 4.68, 95% C.I = (1.54–8.65) with *P* < 0.05, indicating that miR-183-5p can be used for a prediction and/or prognostication of BC. miR-183-5p was found to neither predict low grade tumors vs. non-cancer urine (*P* = 0.4), nor high grade vs. low grade BC urine samples (*P* = 0.1). Additionally, in tissue biopsies, miR-183-5p couldn’t predict BC in the current study (Table 8). We have also conducted a preliminary multivariate analysis based on the data for confounding variables such as age, sex, smoking, stage and grade (Table 8), which normally could enhance the predictive accuracy and clinical applicability of the diagnostic model, while effectively controlling for potential confounding factors that may influence the results according to Zhang et al., 2024 [33]. However, this was not the case and could be due to the need to further expanding the dataset in future studies.

**Table 8:** Univariate and analysis of miR-183-5p in the urine cytology and tissue biopsies

	Parameter	OR	95% C. I		P. value
			Lower	Upper	
Tissue biopsies	Urine	4.68	1.54	8.65	0.05*
	Tissue	1.8	1.1	2.8	0.3
Urine samples	Low grade BC vs. non-cancer control	8.512	3.21	18.09	0.4
	High grade vs. Low grade BC	2.763	0.787	9.701	0.1
Multivariate Analysis	Age	0.875	0.698	1.097	0.247
	Sex	1.000	0.521	0.865	0.523
	Smoker	1.429	0.112	18.298	0.784
	Stage	0.095	0.006	1.575	0.100
	Grade	0.694	0.136	1.032	0.431

BC: Bladder cancer. OR: Odd Ratio, C.I.; Confidence Interval, P value calculated depending on log-linear regression analysis. *P value < 0.05 is significant, **P value < 0.001 is highly significant. Logistic Regression: OR odd ratio, 95% C.I; 95% confidence interval.

### Immunohistochemical findings

**Fig. 3:**
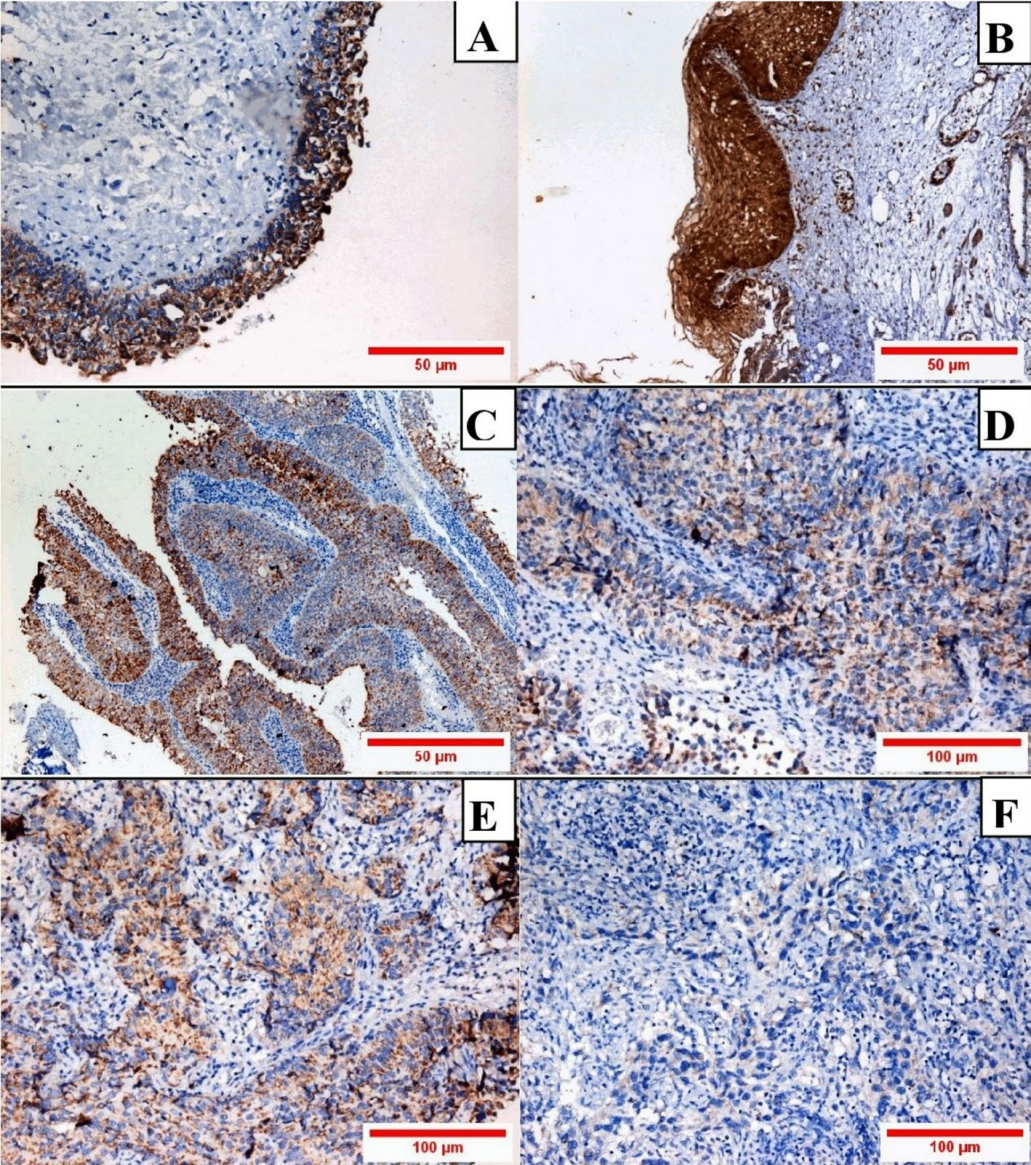
TPM1 protein immunohistochemistry (IHC) expression among the studied cases (IHC for TPM1, DAB, a-b-c = X100, d-e-f = X200). A: Non-cancer control shows moderate cytoplasmic TPM1 staining in the transitional type urothelium. B: Non-cancer control normal squamous lining of the trigone exhibits strong diffuse cytoplasmic TPM1 staining. C. Low grade papillary urothelial carcinoma demonstrates moderate to strong cytoplasmic TPM1 expression. D: Non-Muscle Invasive Squamous Cell Carcinoma (NMIBC) with moderate TPM1 expression. E: High grade Muscle Invasive (MIBC) urothelial Carcinoma with weaker cytoplasmic staining compared to MIBC. F: High grade MIBC negative for TPM1 staining

Overall, Tropomyosin-1 protein (TPM1) evaluation revealed its downregulation in BC tissue biopsies by IHC staining. Surprisingly, benign urothelial cells significantly exhibited the highest TPM1 score in contrast to malignant cells (*P* < 0.0001), with no significant differences between TCC and SCC (*P* > 0.5). Simultaneously, the low-grade and stage BCs significantly expressed higher TPM1 scores compared to the high grade, high stage BC (*P* < 0.001). Similarly, BC with negative lymph nodes showed higher TPM1 values compared to BC with lymph node metastasis (Table 9) (Fig. 3A-F).

In the same context, BC tissues that showed negative HGUC in urine cytology significantly exhibited higher TPM1 scores contrasting BC tissues that presented with positive HGUCs in urine cytology (Table 9).

**Table 9:** Association between the pathological parameters and TPM1 protein expression scores

Characteristic		*N*	TPM1 (IRS) Mean ± SD	*P*. value
Cytology	Negative HGUC	48	2.4 ± 1.3	< 0.001^**^
	Positive HGUC	100	1.8 ± 0.9	
Pathology	Benign (cystitis)	30	6.1 ± 1.9	
	SCC	27	2.0 ± 0.8	> 0.4
	TCC	121	2.0 ± 1.1	
Tumor Grade	GI	16	3.0 ± 0.7	< 0.001^**^
	GII	48	2.7 ± 1.2	
	GIII	84	1.4 ± 0.6	
Tumor Stage	Ta	16	2.5 ± 0.5	< 0.001^**^
	T1	14	2.4 ± 1.2	
	T2	35	2.5 ± 1.2	
	T3	83	1.6 ± 0.9	
Lymph Nodes Metastasis	Not available	10	6.1 ± 1.9	< 0.001^**^
	Negative	56	2.17 ± 1.0	
	Positive	92	1.6 ± 1.0	

IRS: Immunoreactive Score. SQCC: Squamous cell carcinoma. TCC: Transitional cell carcinoma. HGUC: High Grade Urothelial Carcinoma. All parameters are represented as Mean ± SD; the data were analyzed by student t-test between two categories, while were analyzed by one-way ANOVA between multicategories parameters. ** High significant difference with the benign (cystitis) cases * P value < 0.05 is significant, ** P value < 0.001 is highly significant.

Regarding the correlation of the study’s parameters, a significantly positive correlation between TPM1 scores and miR-183-5p expression (*P* < 0.001) with a significantly negative correlation between the BCs grades and both TPM1 score (*P* < 0.001), and miR-183-5p expression (*P* < 0.001) in BC tissue sections. Similarly, BC stage showed a significantly negative correlation with the TPM1 score of expression (*P* < 0.05) as well as a non-significant negative correlation with the expression of miR-183-5p in BC tissues (Table 10).

**Table 10:** Correlation between TPM1 protein expression and miR-183-5p in bladder tissue of studied cases

Parameter			TPM1 Protein expression	miR-183-5p	Tumor Grade	Tumor Stage
Spearman’s rho	TPM1 protein Expression	Correlation Coefficient	1.000	0.416^**^	− 0.598^**^	− 0.352^*^
		Sig. (2-tailed)		0.005	0.000	0.019
	miR-183-5p	Correlation Coefficient	0.416^**^	1.000	− 0.512^**^	− 0.244
		Sig. (2-tailed)	0.005		0.000	0.110
	Tumor Grade	Correlation Coefficient	-0.598^**^	-0.512^**^	1.000	0.502^**^
		Sig. (2-tailed)	0.000	0.000		0.001
	Tumor Stage	Correlation Coefficient	-0.352^*^	-0.244	0.502^**^	1.000
		Sig. (2-tailed)	0.019	0.110	0.001	

Correlation is significant at the 0.01 level (2-tailed). *Correlation is significant at the 0.05 level (2-tailed). **Significant positive correlation between TPM1 protein expression and miR-183-5p (*r* = 0.416, *P* < 0.001). Significant negative correlation between the tumor grade and both TPM1 protein expression (*r*= -0.598, *P* < 0.001) and miR-183-5p (*r*=-0.502, *P* < 0.001) in tissue sections. The tumor stage showed a significant negative correlation with TPM1 protein ex-pression (*r*= -0.352, *P* < 0.05) and non-significant negative correlation with miR-183-5p (*r*=-0.244, *p* > 0.1) in tissue sections.

## Discussion

According to the Global Cancer Observatory (2020) [1], bladder carcinoma (BC) is among the most prevalent cancers in Egypt [34]. MicroRNAs (miRNAs) can be easily isolated from body fluids such as urine and serum of BC patients with minimal invasiveness [15]. The identification of molecular targets involved in tumorigenesis is therefore critical for improving BC diagnosis and treatment. Several miRNAs have been reported to regulate diverse target genes [6].

In this study, we evaluated miR-183-5p expression in 148 urine samples from BC patients and their corresponding tissue biopsies. Our findings demonstrated promising diagnostic performance for miR-183-5p in both urine cytology and tissue samples. Specifically, miR-183-5p was significantly upregulated in the urine of BC patients compared with controls, and its expression increased progressively with tumor grade. These results are consistent with those of Li et al. [35], who reported a significant correlation between serum miR-183-5p levels and BC histological grade. Similarly, Grimaldi et al. [36] and El-Shal et al. [37] observed upregulated miR-183-5p in urine samples from BC patients. In tissue biopsies, we also found significantly higher miR-183-5p expression in BC tissues compared with normal urothelium, consistent with the findings of Wei et al. [38] and Yamada et al. [39]. In addition, El-Shal et al. [37] reported diagnostic sensitivity and specificity of 78.4% and 81.6%, respectively, for urinary miR-183-5p in distinguishing BC from non-BC cases, which is in line with our observations. Importantly, we also found good sensitivity (83.3%) and specificity (61.5%) for differentiating low- from high-grade BC. Logistic regression analysis further underscored the prognostic value of miR-183-5p expression in BC diagnosis and stratification. These findings parallel those of Xu et al. [40], who supported miR-183-5p as a reliable biomarker for the diagnosis and prognosis of lung adenocarcinoma.

In contrast, our immunohistochemical (IHC) analysis revealed downregulation of TPM1 protein expression in BC tissues. Benign urothelium showed the highest TPM1 scores, whereas malignant tissues exhibited significantly lower expression (*P* < 0.0001). Moreover, BC tissues with negative urine cytology for high-grade urothelial carcinoma (HGUC), absence of nodal metastasis, and lower stage and grade expressed higher TPM1 levels compared with tumors exhibiting positive HGUC cytology, nodal metastasis, higher grade, and advanced stage. These results are consistent with Lin et al. [23], who reported TPM1 downregulation in solid tumors including urothelial carcinoma, with expression inversely correlated with tumor grade, muscle invasiveness, and metastasis. Liu et al. [41] also confirmed these findings. Collectively, reduced TPM1 expression appears to indicate poor prognosis. As TPM1 is detectable in urine, its evaluation may represent a promising noninvasive diagnostic tool and a risk stratification marker for BC, as suggested by Humayun et al. [42] and Yan et al. [43].

With respect to the functional role of miR-183-5p, Hu et al. [7] described the miR-183-5p–PNPT1 regulatory axis, which influences apoptosis in BC and may represent a therapeutic target. Our study identified an inverse relationship between miR-183-5p expression and TPM1 expression. Specifically, miR-183-5p was upregulated in both urine and tissue, whereas TPM1 protein was markedly downregulated in BC tissues. This pattern is consistent with the work of Liu et al. [41], who reported upregulated miR-96 and downregulated TPM1 in malignancy. Bioinformatic prediction using TargetScan identified TPM1 as a direct target of miR-183-5p.1 through binding to its 3′UTR, and transfection of T24 cells with miR-183-5p.1 mimics significantly decreased TPM1 mRNA levels. Thus, miR-183-5p suppresses TPM1 expression in vitro.

Recent evidence also emphasizes the importance of determining oncogenic roles in bladder cancer. Li et al. (2023) demonstrated that S100A5 attenuates the efficacy of anti-PD-L1/PD-1 immunotherapy by inhibiting CD8⁺ T cell–mediated anti-tumor immunity [44]. These findings highlight how oncogenic regulators can shape the tumor immune microenvironment and influence therapeutic response. In this context, our study contributes to understanding the oncogenic role of miR-183-5p as an onco-miR, showing its ability to suppress TPM1, a tumor suppressor, and thereby promoting urothelial carcinogenesis. This further underscores why it is important to investigate miRNAs for their potential epigenetic theranostic roles. By characterizing miR-183-5p as an oncogenic promoter that downregulates TPM1, our work supports the concept of miRNA-based strategies as both diagnostic and therapeutic tools in bladder cancer.

At first glance, these in vitro results appear contradictory to our clinical correlation analysis, which showed a significant positive association between miR-183-5p and TPM1 expression in patient tissues. This apparent discrepancy may be explained by the presence of strong upstream regulatory mechanisms in vivo. For example, oncogenic transcription factors could simultaneously drive the expression of both miR-183-5p and TPM1, thereby overriding the direct suppressive effect of miR-183-5p on TPM1 observed under controlled in vitro conditions. Such context-dependent regulation highlights the complexity of tumor biology and underscores the need for further mechanistic studies to clarify this relationship.

Taken together, our findings establish miR-183-5p as an oncogenic promoter (onco-miR) [45]. that directly targets and downregulates TPM1, a tumor suppressor gene, thereby contributing to BC progression. High miR-183-5p expression in tissues and urine, combined with reduced TPM1 expression, highlights the biological and clinical significance of this axis. These results not only support the theranostic potential of miR-183-5p in BC diagnosis, prognosis, and therapy but also identify TPM1 as a key molecular target with implications for disease management.

Limitations and Future Directions: This study, through bioinformatic analysis, cellular experiments, and clinical correlation, strongly proposes the hypothesis that miR-183-5p plays a role in bladder cancer by targeting TPM1. However, all functional validation data currently come from in vitro cell line models. While these data are highly suggestive, in vivo animal experiments are indispensable to conclusively demonstrate the direct causal role of this pathway in bladder cancer development and progression, and to evaluate its potential as a therapeutic target. Building on these findings, further investigation of the miR-183-5p–TPM1 axis could provide valuable insights into its biological and clinical significance. In vivo studies using appropriate animal models could clarify the potential role of this pathway in tumor initiation, progression, and metastasis, and may inform the development of therapeutic strategies. Targeted interventions, such as miR-183-5p inhibitors or TPM1-mimetic approaches, could be explored as potential therapeutic options. Assessing the influence of miR-183-5p on the tumor immune microenvironment, particularly CD8⁺ T cell activity and response to immune checkpoint blockade, might further elucidate its role in modulating anti-tumor immunity. Longitudinal clinical studies in larger patient cohorts, examining urinary and tissue expression of miR-183-5p and TPM1, could support their evaluation as prognostic and diagnostic biomarkers. Additionally, integrating multi-omics analyses may reveal broader regulatory networks involving miR-183-5p and TPM1, and investigating combinatorial biomarker panels could enhance diagnostic precision, risk stratification, and therapeutic monitoring in bladder cancer.

## Data Availability

No datasets were generated or analysed during the current study.
